# Significant Differences in Markers of Oxidant Injury between Idiopathic and Bronchopulmonary-Dysplasia-Associated Pulmonary Hypertension in Children

**DOI:** 10.1155/2012/301475

**Published:** 2012-07-05

**Authors:** Kimberly B. Vera, Donald Moore, English Flack, Michael Liske, Marshall Summar

**Affiliations:** ^1^Division of Cardiology, Department of Pediatrics, Vanderbilt University School of Medicine, Nashville, TN 37232, USA; ^2^Center for Genetic Medicine Research, Children's National Medical Center, Washington, DC 20010, USA

## Abstract

While oxidant stress is elevated in adult forms of pulmonary hypertension (PH), levels of oxidant stress in pediatric PH are unknown. The objective of this study is to measure F_2_-isoprostanes, a marker of oxidant stress, in children with idiopathic pulmonary hypertension (IPH) and PH due to bronchopulmonary dysplasia (BPD). We hypothesized that F_2_-isoprostanes in pediatric IPH and PH associated with BPD will be higher than in controls. Plasma F_2_-isoprostanes were measured in pediatric PH patients during clinically indicated cardiac catheterization and compared with controls. F_2_-Isoprostane levels were compared between IPH, PH due to BD, and controls. Five patients with IPH, 12 with PH due to BPD, and 20 control subjects were studied. Patients with IPH had statistically higher isoprostanes than controls 62 pg/mL (37–210) versus 20 pg/mL (16–27), *P* < 0.01). The patients with PH and BPD had significantly lower isoprostanes than controls 15 pg/mL (8–17) versus 20 pg/ml (16–27), *P* < 0.02. F_2_-isoprostanes are elevated in children with IPH compared to both controls and patients with PH secondary to BPD. Furthermore, F_2_-isoprostanes in PH secondary to BPD are lower than control levels. These findings suggest that IPH and PH secondary to BPD have distinct mechanisms of disease pathogenesis.

## 1. Introduction

It has long been recognized that patients with pediatric idiopathic pulmonary hypertension (IPH) have poor long-term survival. More recently pulmonary hypertension (PH) associated with bronchopulmonary dysplasia (BPD) has been identified as a significant cause of mortality among BPD patients [1, 2]. Few studies have evaluated the mechanisms and optimal treatment of PH due to BPD, resulting in management strategies for these patients which mirror the better studied pharmacologic treatments of IPH. The use of similar therapeutic strategies in these two populations relies on the unproven assumption that the diseases share similar molecular pathophysiologies.

Oxidant stress appears to play a role in the molecular mechanism of adult IPH. Multiple studies measuring F_2_-isoprostanes, a stable marker of oxidant stress resulting from the oxidation of cell-membrane arachidonic acid, have shown adult IPH patients, have higher F_2_-isoprostane levels than do control patients [3, 4]. Elevated F_2_-isoprostane levels suggest enhanced oxidant stress in IPH patients and may also directly contribute to pulmonary vasoconstriction [5]. There are no published data on oxidant stress or F_2_-isoprostane levels in pediatric patients with PH secondary to BPD or IPH. The objective of this study is to measure F_2_-isoprostanes in children with IPH and PH due to BPD and to compare them to normal controls to assess the role of oxidant stress in pediatric populations with PH. We hypothesize that children with IPH and PH due to BPD will have F_2_-isoprostane levels higher than those measured in healthy control subjects. Evidence supporting similar biochemical mechanisms between these pediatric populations with PH would support the practice of utilizing similar therapeutic strategies in these children.

## 2. Materials and Methods

### 2.1. Study Population

All patients who presented to the pediatric catheterization laboratory at Vanderbilt Children's Hospital for evaluation of pulmonary hypertension between December 2007 and December 2008 were approached for participation in the study. Patients were excluded if they had ventricular septal defects; patients with an atrial septal defect or hemodynamically insignificant patent ductus arteriosus were allowed. Other exclusion criteria were pulmonary vein stenosis, valvar stenosis of any kind, aortic arch obstruction, left ventricular dysfunction, active infection of any kind, and autoimmune disease. All catheterizations were performed for clinical reasons in accordance with the standard of care at the Vanderbilt Pulmonary Hypertension Center. 

Two groups of control patients were enrolled. The primary control group was recruited from the general pediatric clinic at Vanderbilt Children's Hospital. Patients without acute or chronic illness who required a routine blood draw for health maintenance were approached for enrollment in the study. In addition, in order to assess the effect of general anesthesia and the general impact of the catheterization on F_2_-isoprostane levels, patients presenting to the pediatric catheterization laboratory for atrial septal defect (ASD) device closure were also approached to participate as controls. Patients undergoing device ASD closure were chosen because they typically do not have elevation of their pulmonary artery pressure and are in good general health. Exclusion criteria for both control groups were a history of prematurity, ventricular septal defect, pulmonary vein stenosis, valvar stenosis of any kind, aortic arch obstruction, left ventricular dysfunction, active infection of any kind, and autoimmune disease. 

### 2.2. Echocardiography

All consenting control subjects recruited from the general pediatric clinic underwent echocardiography to screen for undiagnosed pulmonary hypertension. Right ventricular pressure was assessed by interrogation of the tricuspid regurgitation jet and utilization of the Bernoulli equation. The right atrial pressure was assumed to be 5 mmHg. Any right ventricular pressure measurement of greater than 30 mmHg was deemed elevated. In the absence of tricuspid regurgitation, flattening of the ventricular septum during systole in the parasternal short axis was defined as evidence of elevated right ventricular pressure. The echocardiograms were independently reviewed by two pediatric cardiologists. Evidence of elevated right ventricular pressure found by one or more reviewer excluded a patient from participation as a control subject.

### 2.3. Cardiac Catheterization

At the Vanderbilt Pediatric Pulmonary Hypertension Center, the timing of cardiac catheterization is specific to each type of PH. IPH patients undergo cardiac catheterization with vasodilatory testing at diagnosis and every 3–12 months thereafter depending on their clinical status and changes in therapy. Patients with PH and BPD are not routinely catheterized at diagnosis unless structural abnormalities are suspected such as pulmonary vein stenosis. Our center uses echocardiography to identify and follow elevated pulmonary artery pressure in neonates with BPD in the neonatal ICU and in follow up after discharge from the ICU. Tricuspid regurgitation velocity and systolic flattening of the ventricular septum are the primary echocardiographic features used to assess pulmonary artery pressure. BPD patients with persistent echocardiographic evidence of elevated pulmonary artery pressure are followed in the PH clinic as outpatients and undergo catheterization within 3–6 months if on vasodilator therapy. BPD-PH patients may also undergo catheterization prior to discontinuation of vasodilator therapy.

Consenting participants with PH and the ASD control patients underwent their clinically indicated cardiac catheterization under general anesthesia. All patients underwent a right heart catheterization with directly measured saturations and pressures at the lowest FiO_2_ were required to maintain oxygen saturations of above 95% by pulse oximetry. In patients with an ASD, a catheter was placed across the ASD from the right heart to obtain a pressure in the left atrium and a saturation measurement from a pulmonary vein. A femoral artery sheath was placed in all patients to directly measure the systemic blood pressure and the descending aortic saturation. Pulmonary flows were calculated using the Fick equation with assumed oxygen consumption in all BPD patients, in the IPH patients with an atrial septal defect, and in all the ASD control patients. Thermodilution was used to measure pulmonary flow in IPH patients without an atrial septal defect. All pulmonary flows were indexed to body surface area. Pulmonary vascular resistance (PVR) was calculated in Woods units (WU) and indexed to body surface area. 

### 2.4. Blood Sampling

In control patients recruited from the general pediatric clinic, 5 mLs of study blood was drawn by routine phlebotomy. ASD control patients and PH patients had 5 mls of study blood drawn during the baseline hemodynamic measurements. If patients undergoing catheterization had a pulmonary venous sample obtained, the study blood was taken from a pulmonary vein. Catheterized patients without an ASD had study blood obtained in the descending aorta through the femoral arterial sheath or arterial catheter. All blood samples were obtained before any pulmonary vasodilator testing was performed. All study blood was collected on ice in an EDTA tube and immediately transported to the laboratory for isoprostane analysis. 

### 2.5. F_2_-Isoprostane Analysis

F_2_-isoprostanes were measured using a method pioneered by Drs. Morrow and Roberts [6]. Briefly this involves passing the sample through two Waters Corporation Sep-Pak cartridges to remove much of the unwanted impurities. First one uses a C-18 packing material and the second uses a silica packing material. The final elutionis then esterified with pentafluorobenzyl bromide and silated with bis(trimethylsilyl)trifluoroacetamide before being subjected to GC/MS analysis on an Agilent 5973 inert MSD coupled with and Agilent 6890N Network GC from Agilent Technologies in Wilmington, Delaware.

### 2.6. Statistical Analysis

Study data were collected and managed using the REDCap electronic data capture tools hosted at Vanderbilt University [7]. Data are presented as medians with interquartile ranges (IQR) due to lack of normal distribution. The Mann-Whitney *U* test was performed to determine the statistical significance of the difference between any two groups. The Kruskal-Wallis test was used to analyze differences between all three groups. Categorical variables between groups were assessed with the Fisher's exact test. Spearman's test was used to analyze correlations. A two-tailed *α* of <0.05 was considered statistically significant. Bonferroni correction was not used due to its conservative nature and the small number of comparisons done in this study. SPSS was used to perform the statistical analysis (IBM SPSS statistics, version 20).

This study was approved by the Institutional Review Board of the Vanderbilt University Medical Center.

## 3. Results

We enrolled 5 patients with IPH and 12 patients with PH secondary to BPD. All PH patients approached consented to participate in the study. Twenty controls, including 5 ASD patients and 15 healthy controls from the primary care clinic, consented to participate in the study. Three eligible control patients approached in the primary care clinic refused to participate because they did not have time to undergo echocardiography. All ASD patients approached to participate consented. None of the healthy control subjects had abnormal echocardiograms. One of the 5 IPH patients underwent diagnostic catheterization while the remainder underwent routine follow-up catheterizations. All of the BPD patients were catheterized for purposes of treatment follow-up. The F_2_-isoprostane data could not be obtained in one IPH patient and two controls due to sample problems. 

Table 1 describes the baseline characteristics of the study groups. The median age of control patients did not statistically differ from IPH patients (*P* = 0.99), but those with PH due to BPD were significantly younger than controls (*P* < 0.01). There was no statistical difference in gender distribution between the controls and the two PH groups. While the body mass index (BMI) of the IPH patients did not significantly differ from the controls, the BMI of those with PH due to BPD was significantly less than controls (*P* = 0.01). 

The medical treatments of patients with PH are described in Table 2. There were significantly more IPH patients treated with epoprostenol than patients with PH due to BPD, although this did not reach statistical significance (*P* = 0.06). There was no difference in the number of subjects on bosentan, sildenafil, and home oxygen in the two PH groups.

The hemodynamic data from the cardiac catheterizations are presented in Table 3. The control patients undergoing ASD device closure had normal right ventricular pressure and pulmonary vascular resistance which were significantly lower than in both the IPH group (*P* < 0.01) and the BPD-PH group (*P* < 0.01). The right and left ventricular end diastolic pressures, cardiac index, and baseline FiO_2_ in ASD controls were not different from either PH group. As expected, those undergoing ASD device closure had a larger pulmonary to systemic blood flow ratio than those with IPH (*P* < 0.01) and PH due to BPD (*P* < 0.01). The median right ventricular pressure as a percentage of left ventricular pressure (RVP/LVP ratio) and the median PVR were distinctly lower in those with PH due to BPD compared to those with IPH, but the differences did not quite reach statistical significance. The median right ventricular end diastolic pressure (RVEDP), left ventricular end diastolic pressure (LVEDP), and baseline FiO_2_ were not significantly different among the two groups with PH. A patent foramen ovale was found in 5 patients with PH secondary to BPD, and a moderate atrial septal defect was found in one patient with PH secondary to BPD. One patient with PH secondary to BPD had a patent foramen ovale in addition to a small patent ductus arteriosus. In those with IPH, two patients had small atrial septal defects.

**Figure 1:**
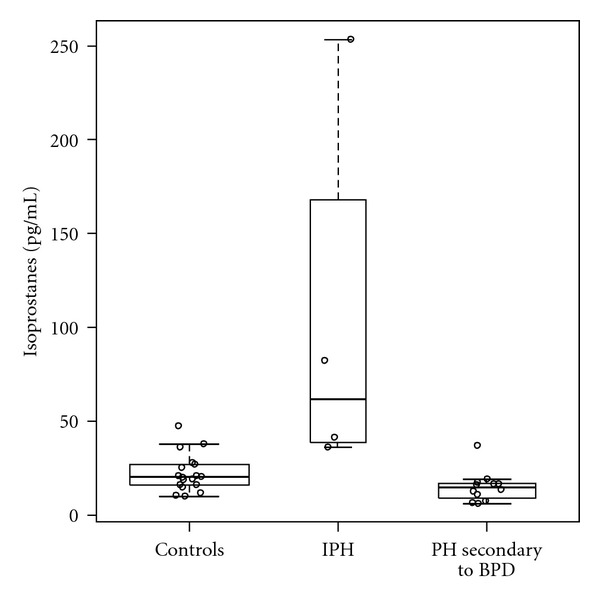
Box plot of plasma F_2_-isoprostanes in IPH, PH due to BPD, and controls. F_2_-Isoprostanes are significantly higher in children with IPH compared to pediatric controls and children with PH secondary to BPD. Plasma F_2_-isoprostanes are statistically lower in children with PH secondary to BPD compared to those with IPH and pediatric controls.

Patients with IPH had significantly higher F_2_-isoprostanes than controls (62 pg/mL (37–210) versus 20 pg/mL (16–27), *P* ≤ 0.01) (Figure 1) . The patients with PH due to BPD had significantly lower F_2_- isoprostanes than controls (15 pg/mL (8–17) versus 20 pg/mL (16–27), *P* = 0.02). F_2_-Isoprostane levels in IPH patients were significantly higher than those with PH secondary to BPD (*P* = 0.002). No correlation was found between F_2_-isoprostane levels and age (*R*_*S*_^2^ = 0.02) or between F_2_-isoprostane levels and BMI (*R*_*S*_^2^ = 0.09) in the study cohort. Among all PH patients, no correlation was found between F_2_-isoprostanes and RVP/LVP ratio (*R*_*S*_^2^ = 0.02) or any other hemodynamic measure. When analyzing only BPD patients with RVP/LVP >50%, those with PH due to BPD still had lower F_2_-isoprostanes ((8.7 pg/mL (6.3–12.7)) than controls (*P* ≤ 0.01) and those with IPH (*P* = 0.02). No significant difference was found between the F_2_-isoprostane levels of the control subjects undergoing ASD device closure and those undergoing routine phlebotomy (*P* = 0.95). Similarly, the F_2_-isoprostanes drawn from the pulmonary veins were not significantly different from those drawn from the descending aorta among those with PH due to BPD (*P* = 0.09) or among those with IPH (*P* = 0.44).

Both the IPH and BPD-PH groups had one outlier with a high F_2_-isoprostane level. The BPD patient with the high isoprostane value had only mildly elevated pulmonary artery pressures with a RVP/LVP ratio of .38 and a PVR of 3.6 WU and was only being treated with sildenafil and home oxygen. The IPH patient with the high isoprostane value had similar hemodynamics to the other IPH patients with a RVP/LVP ratio of .83, a PVR of 17.1 WU, a RVEDP of 10 mmHg, and a LVEDP of 10 mmHg. This IPH patient was on epoprostenol and sildenafil similar to the remaining IPH patients but was the only one on bosentan. When repeating the analysis without the two outlier, the F_2_-isoprostanes of the IPH group are still significantly higher than the BPD-PH group (*P* = 0.01), and the control F_2_-isoprostanes are still significantly lower than the IPH group (*P* = 0.02) and higher than the BPD-PH group (*P* < 0.01).

## 4. Discussion

F_2_-isoprostanes are elevated in pediatric patients with IPH but not in those with PH secondary to BPD. In fact, F_2_-isoprostanes in the BPD-PH group seem to be lower than controls. Anesthesia and the general effect of the catheterization did not appear to influence F_2_-isoprostane levels as there was no difference between the ASD controls and the clinic controls. To our knowledge, this is the first time F_2_-isoprostane levels have been studied in pediatric patients with IPH and PH secondary to BPD. Different F_2_-isoprostane levels suggest that IPH and PH secondary to BPD have distinct molecular pathophysiologies with different degrees of chronic oxidant injury. This suggests that these entities may be amenable to different pharmacologic approaches. The finding of elevated F_2_-isoprostanes in children with IPH is consistent with the elevated levels previously reported in the adult populations with IPH [3]. F_2_-isoprostanes have been shown to have a direct role in producing pulmonary vasoconstriction by the activation of thromboxane receptors and increasing the production of potent vasoconstrictors such as thromboxane A_2_ and endothelin 1 [3, 8]. Our finding of elevated circulating F_2_-isoprostane levels in pediatric patients with IPH suggests enhanced oxidant stress in these patients which may directly contribute to pulmonary vasoconstriction.

The low levels of F_2_-isoprostanes in PH secondary to BPD was an unexpected finding. Two groups have previously shown elevated levels of F_2_-isoprostanes in premature infants in the first weeks of life [9, 10]. Impaired and disordered angiogenesis and resultant impaired alveolarization due at least in part to oxidant damage is thought to underlie much of the BPD phenotype [11]. The natural history of oxidant stress in premature infants with or without PH due to BPD is unknown. In this study, there was no difference in months on treatment between the IPH group and the BPD-PH group suggesting the two cohorts are at reasonably similar points in disease time course. Even if the F_2_-isoprostane levels are elevated early in the course of PH secondary to BPD, the low levels we found in these established BPD-PH patients is in marked contrast to the elevated levels we found in IPH patients at a similar point in disease course. 

The etiology of low levels of F_2_-isoprostanes in children with PH secondary to BPD is unknown. F_2_-isoprostanes are formed by the free-radical-induced peroxidation of arachidonic acid in cell membranes [4, 12]. This would suggest that PH secondary to BPD does not generate the oxidant stress seen in IPH at the molecular level and/or that BPD enhances compensatory mechanisms to scavenge free radicals. An alternative possibility would be the preferential production of other isoprostane molecules from arachidonic acid, such as E_2_ and D_2_ isoprostanes, in children with BPD-associated PH. Polyunsaturated fatty acids such as linoleic acid, DHA, and EPA may be oxidized to form isoprostane like molecules more efficiently than arachidonic acid [12]. Children with PH due to BPD may have an unknown mechanism to encourage oxidation of these polyunsaturated fats over arachidonic acid. This is another potential explanation of the low F_2_-isoprostane levels in children with BPD-associataed PH, although there is no data on this possibility. Another alternative is inhibition of F_2_-isoprostane formation by very high oxygen tension with diversion to isofuran production; however, those with BPD-associated PH in this study were not on significantly higher FiO_2_ than the other groups [13]. In fact, all of the groups were breathing a FiO_2_ of near 21% making hyperoxic suppression of F_2_-isoprostanes very unlikely. The inevitable pO2 difference between the pulmonary venous samples and systemic venous samples did not appear to influence F_2_-isoprostane levels as there was no difference in the F_2_-isoprostane levels between the ASD controls, who all had pulmonary venous samples, and the clinic controls, who all had systemic venous samples. Regardless of any potential mechanism to lower F_2_-isoprostane levels below normal controls, this study strongly supports the absence of a high level of uncompensated oxidant stress in this population of children with PH secondary to BPD. 

The limitations of this study include the fact that the patients with PH due to BPD are younger and have a lower BMI than both the controls and those with IPH. The age difference is difficult to remedy as IPH typically presents later in childhood while PH due to BPD is a disease of infants and toddlers. If the patient survives infancy, PH secondary to BPD tends to improve or even resolve with age leaving few older children with active disease to study [2]. The lower BMI in the PH due to BPD is likely a function of both the younger age in this group and the commonly seen feature of failure to thrive early in life in patients with BPD. The absence of correlation of age or BMI with F_2_-isoprostane level suggests the age and BMI differences do not explain the difference in F_2_-isoprostanes seen in this study. 

 Another limitation of the study is the different PH severity among the IPH group and those with PH due to BPD. If infants survive the initial malignant phase of PH secondary to BPD, pulmonary artery pressures tend to decrease over time [2]. This natural history of improvement in PH due to BPD explains the lower pulmonary vascular resistance and RVP/LVP in those with PH secondary to BPD compared to those with IPH. The fact that no correlation exists between hemodynamic measures of elevated pulmonary pressures and F_2_-isoprostanes suggests the F_2_-isoprostane difference between IPH and PH due to BPD is not caused by the difference in PH severity. Similarly, analysis of only the BPD PH patients with RVP/LVP >50% continues to show significantly lower F_2_-isoprostanes than IPH patients and controls. While clinical function data, such as New York Heart Association class, was not collected due to the difficulty in applying these measures to infants and toddlers, normal ventricular filling pressures, and normal cardiac outputs in both groups demonstrate similar stable hemodynamic states despite the difference in PH severity. A greater percentage of those with IPH were on treatment with epoprostenol when compared to those with PH due to BPD. Evidence exists that this drug lowers F_2_-isoprostane levels in patients; thus, this bias would act to lessen the difference between IPH patients and the other 2 study groups [3]. Finally, it would be optimal to increase the number of patients in the IPH group, but the rarity of this disease in children makes obtaining larger numbers difficult. 

## 5. Conclusion

We found that pediatric patients with IPH have elevated F_2_-isoprostane levels while children being followed for PH secondary to BPD have low F_2_-isoprostane levels. This marked difference in oxidant stress suggests each disease has a unique pathophysiology. Future studies are needed to better elucidate these differences thereby leading to better targeted therapies for pediatric patients with a broad spectrum of pulmonary hypertensive diseases. 

## Figures and Tables

**Table 1:** Subjects' demographics.

	Control	IPH	PH due to BPD	*P* value
*N*	18	4	12	
Age^§^ (years)	7.5 (3.8–15.3)	11 (3–16.8)	2 (1–2.8)	<.01^∗^
Gender (# male)	10	2	10	.63**, .12^†^, .25^*¶*^
BMI^§^ (kg/m^2^)	17.9 (16.6–25.2)	23.1 (15.4–27.1)	16.1 (15.7–16.7)	.02^∗^
Race (# Caucasian)	10	4	8	.14**, .41^†^, .27^*¶*^

^∗^ *P* value based on the Kruskal-Wallis test.

^∗∗^ *P* value based on the Fisher's exact test of control versus IPH.

^†^ *P* value based on the Fisher's exact test of control versus PH due to BPD.

^¶^ *P* value based on the Fisher's exact test of IPH versus PH due to BPD.

^§^Data expressed as median (IQR).

**Table 2:** Medical therapy of pulmonary hypertension patients.

	IPH	PH due to BPD	*P* value
N	4	12	
Home	1	8	.13^∗^
Oxygen			
(No. of patients)			
Epoprostenol	3	2	.06^∗^
(No. of patients)			
Bosentan	1	1	.52^∗^
(No. of patients)			
Sildenafil	3	11	.45^∗^
(No. of patients)			
Months on	17.5 (4–27.5)	20 (12–29)	.99^∗∗^
Therapy^†^			

*P value based on Fisher's exact test.

**P values based on Mann-Whitney *U* test.

^†^Data expressed as median (IQR).

**Table 3:** Hemodynamics of subjects in catheterization laboratory^¶^.

	IPH	PH due to BPD	ASD controls	*P* value

				IPH versus BPD
*N*	4	12	5	
RVP/LVP (%)	83 (80–1.1)	46 (38–59)	.24 (.22–.25)	.08^∗^
Pulmonary vascular resistance (WUs)^†^	17.2 (16.2–19.6)	4 (3.5–5.4)	1.4 (1.1–1.6)	.08^∗^
Mean PAP^††^ (mmHg)	61 (53–63)	25 (22–36)	17 (14–19)	.08^∗^
RVEDP^‡^ (mmHg)	10 (8–10)	7.5 (6.3–9)	8 (7.5–8.5)	.26^∗^
LVEDP^6^ (mmHg)	8 (8–9)	8 (7–9)	10 (8–10)	.99^∗^
Cardiac index (L/min/m^2^)	3.6 (3.1–3.6)	3.6 (3.5–3.8)	3.0 (2.7–3.5)	.99^∗^
Qp : Qs^§^	.87 (0.8-.94)	1.0 (1.0-1.0)	2.1 (1.6–4.1)	.53^∗^
Baseline FiO_2_(%)	21 (21–26)	21 (21-22)	21 (21–21)	.52^∗^
ASD (# patients)	2	7	5	.62^∗∗^

^∗^Value based on Mann-Whitney *U* test.

^∗∗^Value based on Fisher's exact test.

^†^Indexed to body surface area.

^††^Pulmonary artery pressure.

^‡^Right ventricular end diastolic pressure.

^‡‡^Left ventricular end diastolic pressure.

^§^Ratio of pulmonary to systemic blood flow.

^¶^Interval data expressed as median (IQR).

## Abbreviations

PH: Pulmonary hypertension
IPH: Idiopathic pulmonary hypertension
BPD: Bronchopulmonary dysplasia
ASD: Atrial septal defect
PVR: Pulmonary vascular resistance
WU: Woods units
IQR: Interquartile range
RVP/LVP: Right ventricular pressure as a percentage of left ventricular pressure
RVEDP: Right ventricular end diastolic pressure
LVEDP: Left ventricular end diastolic pressure.
